# Measuring readiness for disaster response in physiotherapy education: development and validation predisposition assessment tool

**DOI:** 10.3389/fpubh.2026.1814454

**Published:** 2026-06-01

**Authors:** Maria da Lapa Rosado, Cláudia Silva, Cristina dos Santos Cardoso de Sá

**Affiliations:** 1 Escola Superior de Saúde do Alcoitão, Departamento de Fisioterapia, Santa Casa da Misericórdia de Lisboa, Lisboa, Portugal; 2 Escola Superior de Saúde do Alcoitão, Departamento de Investigação Saúde e Sociedade, Santa Casa da Misericórdia de Lisboa, Lisboa, Portugal; 3 SPRINT-Sport Physical Activity and Health Research & Innovation Center, Lisbon, Portugal

**Keywords:** disaster preparedness, emergency, disaster and humanitarian action (EDHA), health workforce development, perceived competence, physiotherapy education, public health preparedness, readiness–competence gap

## Abstract

The increasing frequency of emergencies, disasters, and humanitarian crises underscores the need for a health workforce adequately prepared to respond across disaster management continuum. Within this context, physiotherapists play an essential role in post-disaster care, yet evidence on students’ readiness for emergency, disaster, and humanitarian action (EDHA) remains limited. This study aimed to develop and content-validate an assessment tool and examine physiotherapy students’ predisposition, perceived competence, and readiness to assume functional roles in EDHA contexts. A two-phase study was adopted. Phase 1 comprised instrument development, including a 12-item questionnaire organized into three conceptual domains assessing predisposition, perceived competence, and readiness to assume functional roles in EDHA contexts. Content validation was performed using a two-round expert panel process with 12 experts, and pre-testing. Phase 2, a cross-sectional design, involved administering the final questionnaire to 126 undergraduate physiotherapy students from six Portuguese higher education institutions. The instrument demonstrated excellent content validity (S-CVI/Ave = 0.98). Although a majority of students reported interest in EDHA contexts (61.9%), only 35.7% felt adequately prepared. Results from the assessment tool showed high self-reported interpersonal preparedness, with 96.8% of students rating empathy and 92.9% collaboration as “good” or “very good.” In contrast, 57.2% reported positive ratings for leadership. A substantial competence gap was observed in technical domains: 60.3% of participants considered themselves not or only slightly capable of delivering specialized rehabilitation training, and 42.1% reported low confidence in training peers to identify rehabilitation needs. Similarly, low perceived competence was reported in psychological first aid and burn management. Predisposition to work in disaster contexts did not differ significantly across academic years [ESSAlcoitão: χ^2^(2) = 0.287, *p* = 0.866; other institutions: χ^2^(2) = 0.200, *p* = 0.905]. Furthermore, 64.3% of students considered curricular preparation insufficient. By providing a validated instrument that captures a composite construct of predisposition, perceived competence, and functional role readiness, this study offers a tool for monitoring educational needs and evaluating the impact of curricular reforms. The identified readiness–competence gap reinforces the urgency of integrating structured, competency-based EDHA training within undergraduate physiotherapy curricula, thereby strengthening public health preparedness and contributing to more resilient health systems in the face of increasingly frequent emergencies and disasters.

## 1 Introduction

In recent decades, disasters of natural and anthropogenic origin have increased in frequency, complexity, and socioeconomic impact, contributing to growing global risk exposure (1). These events generate substantial social, psychological, and public health consequences, as reflected in global monitoring of health-related Sustainable Development Goal indicators (2). Strengthening coordinated action across the disaster risk management continuum—prevention, preparedness, response, and recovery—is therefore essential to safeguard population health and reinforce health system resilience. The scope of rehabilitation needs emerging from these scenarios is substantial and persistent, resulting in a high demand for specialized care aimed at preserving and restoring the functionality of affected individuals (3). In this context, physiotherapy is recognized as a central component of emergency and disaster responses, given the high prevalence of musculoskeletal, neurological, and respiratory injuries, and the importance of interventions to prevent complications, manage pain, and restore functional autonomy (4, 5). Furthermore, international frameworks emphasize the integration of rehabilitation within coordinated emergency health responses to ensure continuity of care across all phases of disaster management (6).

The relevance of rehabilitation professionals’ participation has been reinforced by international guidelines and professional organizations’ documents, emphasizing that the physiotherapist’s contribution extends from the acute phase to long-term rehabilitation (7, 8). In the acute phase, this contribution includes initial stabilization, prevention of complications associated with immobility, optimization of mobility, and pain management. In subsequent phases, it encompasses rehabilitation of traumatic injuries, adaptation and training with orthoses and prostheses, strengthening and functional training, as well as respiratory physiotherapy using specific techniques to restore pulmonary function (7–9). In coordination with multidisciplinary teams, physiotherapy also contributes to psychosocial well-being by reducing post-traumatic stress through structured physical activity (8).

This framework is also reflected in operational requirements within international Emergency Medical Teams, in which the inclusion of rehabilitation professionals, with mandatory participation of physiotherapists in the most complex teams, aims to ensure rehabilitation care ranging from outpatient levels to surgical inpatients with high dependency (10, 11). This highlights the need for availability and preparedness of physiotherapists to integrate emergency teams across different contexts and countries with varying income levels (12).

Despite this recognition, significant gaps remain in training and specific preparedness for practice in emergency, disaster, and humanitarian action (EDHA) contexts. Several undergraduate physiotherapy curricula address the subject in an incipient and unsystematic way, raising questions about students’ predisposition (availability and intention) and perceived competence to intervene in unpredictable and highly demanding environments. Factors such as lack of prior experience, uncertainty regarding roles within teams, and doubts about technical and transversal skills may condition readiness for effective involvement (13–15). Identifying these barriers and motivators is crucial to guiding curriculum revisions and capacity-building strategies.

From an evidence perspective, although guiding documents exist regarding the role of physiotherapists throughout the disaster continuum, there is a scarcity of methodologically robust studies that characterize the predisposition of physiotherapists and physiotherapy students to participate in EDHA responses. This scarcity contrasts with the situation observed in other health professions, where more comprehensive levels of disaster preparedness and performance are reported (16–18). Such misalignment underscores the need for specific, valid, and reliable instruments to quantify predisposition, perceived preparedness, and relevant competencies, as well as to identify training gaps with direct implications for public health and the resilience of health systems.

Therefore, the present study aimed to develop and validate the content of a questionnaire designed to assess the predisposition of physiotherapy students to work in emergency, disaster, and humanitarian action scenarios (Phase 1); to characterize and to identify their perceived preparedness and self-assessed competencies (Phase 2).

## 2 Methods

### 2.1 Study design

This study followed a two-phase design combining instrument development and a cross-sectional survey.

Phase 1 – Instrument development and content validation: A questionnaire designed to assess physiotherapy students’ predisposition to work in EDHA contexts was developed and content-validated using a two-round expert panel consultation (Delphi-informed process). Subsequently, pre-testing with physiotherapy students was conducted to evaluate item clarity, relevance, and comprehensibility.

Phase 2 – Cross-sectional survey: The final version of the questionnaire was administered to a sample of undergraduate physiotherapy students from Portuguese higher education institutions. This phase explored differences in predisposition, perceived competence, and readiness across academic years and institutions.

This design was considered appropriate for describing attitudes and perceptions without manipulation of variables, allowing objective, measurable data to be collected and analyzed (19, 20). The descriptive nature of the study enabled a comprehensive understanding of the phenomena, while the cross-sectional approach allowed the capture of a specific moment in time (21).

### 2.2 Phase 1 – Questionnaire development and content validation

#### 2.2.1 Instrument development

The questionnaire titled “*Physiotherapy Students’ Predisposition to Work in Disaster, Emergency and Humanitarian Action Contexts*” was developed in the academic year 2023/2024 by a group of final-year students from the Escola Superior de Saúde de Alcoitão. The instrument aimed to evaluate key aspects related to EDHA readiness, including previous training, perceived technical and interpersonal competencies (soft skills), knowledge of disaster-related topics, and willingness to engage in these contexts.

Item generation was conducted during Phase 1 of the study as part of the instrument development process. An initial pool of items was developed by the research team based on a comprehensive review of the literature related to physiotherapy roles and competencies in emergency, disaster, and humanitarian action contexts, as well as relevant international guidelines (3, 5, 11, 22, 23). This literature-informed process resulted in the preliminary version of the questionnaire, which is provided as Supplementary Material 1. This version was subsequently submitted to a formal two-round expert panel consultation for content validation. The development process followed established recommendations for questionnaire design and validation (24).

The questionnaire was developed using Microsoft Forms and comprised five sections: (1) informed consent; (2) sociodemographic and personal background; (3) knowledge and interest in EDHA contexts; (4) self-assessment of technical and interpersonal competencies; and (5) predisposition and influencing factors. The overall structure of the assessment tool developed in this study is summarized in Table 1. Sections 3–5 correspond to the assessment tool developed in this study and included 12 main items organized into three conceptual domains: knowledge and interest in EDHA contexts, self-assessed technical and interpersonal competencies, and predisposition and influencing factors related to engagement in EDHA contexts.

**Table 1:** Structure of the *Physiotherapy Students’ Predisposition to Work in Disaster, Emergency and Humanitarian Action Contexts* questionnaire developed in Phase 1.

Section/domain	Purpose	Number of items	Example content
Sociodemographic and background information	Characterize participants’ academic and personal profile	Several descriptive items	Gender, age, academic year, previous BLS training
Knowledge and interest in EDHA contexts	Assess familiarity with disaster preparedness and interest in EDHA activities	3 items	Knowledge of emergency plans; interest in EDHA
Self-perceived competencies	Evaluate perceived technical and interpersonal competencies relevant to disaster response	5 items	Burn management, immobilization, teamwork
Predisposition and influencing factors	Assess willingness to engage in disaster response and factors influencing participation	4 items	Willingness to participate; barriers and motivators

The assessment tool analyzed in this study corresponds to Sections 3–5 and includes 12 main items organized into three conceptual domains.

The section on competencies was informed by the Disaster Management Report from World Physiotherapy (11) and included five-point Likert-type scales to assess participants perceived competence and preparedness in key intervention areas relevant to disaster response. For technical competencies, response options ranged from *“not competent” to “highly competent,”* while for functional roles, the scale ranged from *“not capable” to “highly capable.”* Soft skills were assessed using a five-point scale ranging from *“very poor” to “very good.”* Soft skills items were selected and discussed by the research team based on attributes identified in the literature as essential for health professionals working in disaster settings (25).

Some items were presented using matrix-style formats that assessed multiple competencies within the same question, and several questions incorporated response-dependent follow-up questions displayed according to participants’ responses. Depending on the item, different response formats were used, including dichotomous (yes/no) responses, multiple-choice options, Likert-type rating scales, and open-ended responses. The questionnaire was originally developed in Portuguese. The full Portuguese version of the instrument is provided in Supplementary Material 2, with an English translation available for comprehension purposes only.

#### 2.2.2 Content validation

Content validation was performed using an expert panel. A total of 12 experts participated in the process, which was conducted over two rounds with the same panel. The panel included physiotherapy educators and clinicians with demonstrated experience or expertise in EDHA contexts. According to established methodological guidance, expert panels do not require a fixed number of participants, and panels ranging from 10 to 18 experts are considered adequate to achieve consensus when participants are purposefully selected for their expertise (26, 27). The size of the panel in this study was therefore deemed appropriate to ensure diverse and informed input during the content validation process.

Validation rounds were conducted online to maximize convenience and preserve respondent anonymity, which enhances honest feedback in open-ended and rating tasks (27). Experts evaluated each item on a 5-point Likert-type scale (1 = strongly disagree to 5 = strongly agree without reservations) to rate each item’s relevance and clarity.

#### 2.2.3 Data analysis

To assess content validity, the Content Validity Index (CVI) was calculated for each item (I-CVI) and overall (S-CVI/Ave), by dividing the number of experts rating an item as 4 or 5 by the total number of experts (28, 29, 30). Items with I-CVI ≥ 0.78 and an overall S-CVI/Ave ≥ 0.80 were considered valid. The final version of the instrument reached an S-CVI/Ave of 0.98, with I-CVIs ranging between 0.875 and 1.00. To finalize the content validation process and to assess clarity and comprehension, a pre-test was conducted with 12 physiotherapy students and recent graduates. After completing the questionnaire, participants were interviewed using a structured comprehension guide form. Qualitative feedback was obtained through structured cognitive interviews conducted after questionnaire completion. Interviews were audio-recorded with participants’ consent and documented using structured comprehension forms. The recordings were reviewed by the research team and analyzed using a qualitative descriptive approach to identify recurrent issues related to item clarity, wording, relevance, redundancy, and adequacy of response options. Suggested modifications were discussed within the research team until consensus was reached, leading to minor revisions in item phrasing, restructuring of selected questions, and refinement of response categories prior to large-scale administration. Two researchers independently reviewed the audio recordings and structured notes. Interviews lasted approximately 10–15 min. The purpose of the pre-test was to identify major comprehension and relevance issues.

### 2.3 Phase 2 – Questionnaire administration and data collection

#### 2.3.1 Participants and sampling

**Figure 1:**
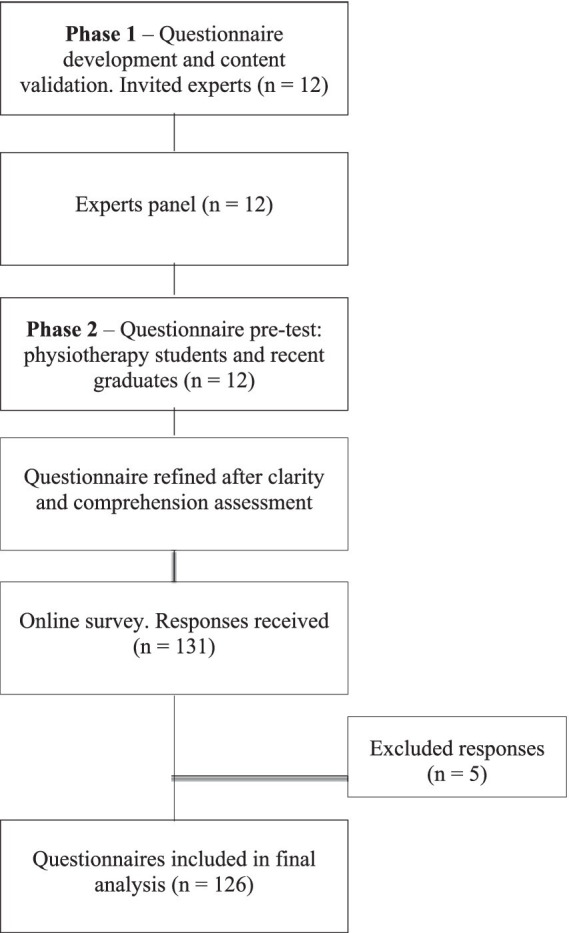
Flow diagram of the study design and participant inclusion across the two phases of the study, including questionnaire development, content validation, pre-testing, and final survey responses.

A total of 131 responses were initially obtained. After screening eligibility criteria, five responses were excluded because they did not meet the predefined inclusion criteria. The final sample consisted of 126 undergraduate physiotherapy students from six Portuguese higher education institutions, recruited through a non-probabilistic convenience sampling approach (see Figure 1).

- Escola Superior de Saúde de Alcoitão,
- Universidade Fernando Pessoa,
- Escola Superior de Saúde Atlântica,
- Universidade do Algarve,
- Escola Superior de Saúde da Cruz Vermelha Portuguesa, and
- Escola Superior de Saúde Egas Moniz.

Inclusion criteria were: (i) enrolment in a physiotherapy undergraduate program (1st to 4th year); (ii) informed consent to participate voluntarily; and (iii) full questionnaire completion. Exclusion criteria included incomplete responses, duplications, and incoherent or automated answers that might compromise data quality (31).

#### 2.3.2 Data collection procedure

Data collection took place between March and April 2025. The questionnaire was distributed digitally through Microsoft Forms via institutional channels and student networks. Participation was anonymous and voluntary. Prior to accessing the questionnaire, participants were presented with an informed consent form outlining the study’s objectives, the nature of data collection, and their right to withdraw at any time without penalty.

#### 2.3.3 Ethical considerations

The study design was reviewed and approved by the Ethics Committee of Escola Superior de Saúde do Alcoitão (Project no. 32/2024-25). The study was classified as minimal risk and conducted using an anonymous, non-invasive, and voluntary online questionnaire administered via Microsoft Forms. Informed consent was obtained electronically from all participants prior to their participation. Data were collected anonymously and handled in accordance with applicable data protection regulations, ensuring confidentiality and restricted access to the research team.

#### 2.3.4 Data analysis

Quantitative data were analyzed using IBM SPSS Statistics (version 30.0; IBM Corp., Armonk, NY, United States).

The analysis followed three steps.

First, descriptive statistics were used to characterize the sample. Absolute and relative frequencies were calculated for categorical variables, including gender, academic year, academic institution, previous Basic Life Support (BLS) training, and previous disaster experience. Means and standard deviations were calculated for continuous variables derived from the questionnaire dimensions. Data from ESSAlcoitão were analyzed separately because this institution represented the majority of the sample.

Second, bivariate analyses were conducted to explore associations between categorical variables, including gender, academic year, academic institution, previous Basic Life Support (BLS) training, previous disaster experience, and students’ predisposition to work in EDHA contexts. Particular emphasis was placed on examining the relationship between students’ predisposition to work in EDHA contexts and their academic year. Chi-square tests were used to examine the relationship between students’ predisposition to work in EDHA contexts (yes/maybe/no) and their academic year (1st–2nd vs. 3rd–4th year) within each institutional group (ESSAlcoitão vs. other institutions).

Three composite dependent variables were defined based on the questionnaire dimensions: soft skills, professional competencies, and functional capacities in disaster response roles. Composite scores were calculated as the mean of the items within each domain (i.e., the sum of item scores divided by the number of items), resulting in scores ranging from 1 to 5, with higher values indicating higher perceived competence. Each score corresponded to the mean of the items belonging to the respective dimension. Internal consistency of the items within each dimension was assessed using Cronbach’s alpha coefficients prior to inferential analyses to evaluate the appropriateness of aggregating items into composite scores.

Third, analysis of covariance (ANCOVA) models were performed to examine the effect of institution (ESSAlcoitão vs. other institutions), included as a fixed factor, on domain scores while controlling for academic year as a covariate, given the unequal distribution of students across academic years between institutions.

Prior to inferential analyses, assumptions of normality and homogeneity of variances were assessed using the Shapiro–Wilk and Levene tests, respectively. Effect sizes were reported using Cohen’s *d* for *t*-tests and partial eta squared (*η*^2^) for ANCOVA. Statistical significance was set at *p* < 0.05.

## 3 Results

The development and application of the questionnaire followed a sequential two-phase process. The flow of the study phases and participant inclusion is presented in Figure 1.

### 3.1 Phase 1 – Instrument development

The panel of experts comprised 12 physiotherapists with extensive professional and academic experience, providing both breadth and depth of expertise. Nine participants were faculty members, while three had direct involvement in disaster and humanitarian action contexts. The group was highly qualified, with academic degrees ranging from bachelor’s to habilitation and a mean of 27 years of professional experience. Notably, two experts had specific training in disaster management and three were members of non-governmental organizations, further reinforcing the appropriateness of the panel for validating an instrument intended for this field.

In the first evaluation round, the questionnaire achieved an overall Content Validity Index (CVI) of 0.89, exceeding the established cut off 0.80. Nevertheless, several items presented partial CVIs below this threshold (minimum of 0.67), highlighting areas requiring refinement. Although the majority of responses assigned the maximum score (72.2%), the variability in item-level evaluations suggested that some questions lacked clarity or precision. These findings underscored the necessity of revising the initial version to ensure semantic accuracy and contextual relevance.

Revisions included eliminating items deemed redundant or irrelevant, splitting complex questions, and introducing linguistic refinements to improve clarity and comprehension. For example, the item initially phrased as “*Do you know the probability of disaster occurrence in your residential area?*” was reformulated to “*Do you know whether your residential area is considered a risk zone?*” to improve conceptual precision. Likewise, an item combining experience and interest in volunteering was split into two separate questions and specified as humanitarian volunteering in order to reduce ambiguity. After these adjustments, the second round demonstrated marked improvement: the overall CVI increased to 0.98, with all items surpassing the minimum threshold and ranging between 0.88 and 1.00. Importantly, response consistency also improved, with 93.8% of ratings at the maximum level and only negligible proportions of lower scores. These results suggest that the refinements successfully enhanced the instrument’s clarity, coherence, and relevance. Table 2 summarizes the Content Validity Index (CVI) results obtained in both expert consultation rounds.

**Table 2:** Content validity results across the two expert consultation rounds.

Domain/Section	CVI round 1	CVI round 2
Sociodemographic section	0.94	0.98
Disaster context knowledge	0.87	0.92
Interpersonal competencies	0.86	0.96
Predisposition to engage in EDHA	0.92	0.98
Overall S-CVI/Ave	0.89	0.98

CVI, Content Validity Index; S-CVI/Ave, scale-level content validity index calculated as the average of item-level CVIs.

Section-wise analysis confirmed this trend, showing consistent improvement across all domains. The sociodemographic section rose from 0.94 to 0.98; the disaster context section from 0.87 to 0.92; the interpersonal skills section from 0.86 to 0.96; and the predisposition section from 0.92 to 0.98. This progression reinforces the robustness of the final version, both in its overall structure and in the representativeness of each specific domain.

The pre-test, conducted with 12 physiotherapy students and recent graduates, further strengthened evidence of the questionnaire’s adequacy. Overall, participants demonstrated good comprehension of the items, as reflected in responses recorded in structured comprehension forms and follow-up interviews. In addition to confirming item relevance, qualitative feedback highlighted issues related to wording clarity, perceived overlap between selected questions, adequacy and completeness of response options, and preferences regarding item structure and formatting. This feedback informed minor linguistic refinements, reorganization or splitting of selected items, consolidation of overlapping questions, and addition or removal of response categories to improve conceptual precision and reduce ambiguity. For example, options such as “prefer not to respond” were introduced in sociodemographic items, combined questions on humanitarian volunteering were separated into distinct items addressing interest and experience, and terminology was refined to align with international professional frameworks. These adjustments enhanced clarity, usability, and content relevance without altering the conceptual domains of the instrument. Together with the results of the expert validation process, these findings support the accessibility and applicability of the questionnaire for use in broader samples.

### 3.2 Phase 2: Questionnaire application

#### 3.2.1 Participant characteristics

A total of 126 undergraduate physiotherapy students from six Portuguese higher education institutions participated in Phase 2. Most respondents were female (76.2%) and aged between 20 and 22 years (57.9%). Students from all academic years were represented, with the highest proportion enrolled in the fourth year (37.3%). The majority attended the Escola Superior de Saúde do Alcoitão (73.8%). Table 3 summarizes the sociodemographic characteristics.

**Table 3:** Sociodemographic and academic characteristics of the participants (*n* = 126).

Variable		*n* (%)
Gender	Female	96 (76.2)
	Male	30 (23.8)
Age	≤19 years	18 (14.3)
	20–22 years	73 (57.9)
	≥23 years	35 (27.8)
Academic Year	1st year	22 (17.5)
	2nd year	26 (20.6)
	3rd year	31 (24.6)
	4th year	47 (37.3)
Academic Institutions	ESSAlcoitão	93 (73.8)
	Other institutions	33 (26.2)
Previous BLS training	Yes	19 (15.1)
	No	107 (84.9)
Previous disaster experience	Yes	33 (26.2)
	No	93 (73.8)

#### 3.2.2 Predisposition and perceived preparedness for EDHA

As shown in Table 4, only 15.1% of participants reported holding Basic Life Support (BLS) certification, although 79.4% expressed interest in obtaining it. Knowledge of local emergency preparedness was limited, with 87.3% unaware of their municipality’s emergency plan and 81.7% not knowing how to access it.

**Table 4:** Students’ predisposition and perceived preparedness for emergency, disaster and humanitarian action (EDHA).

Item	*n* (%)
Interest in EDHA	78 (61.9)
Willingness to participate	58 (46.0)
Uncertain about participation	40 (31.7)
Unwilling to participate	28 (22.2)
Perceive themselves as prepared	45 (35.7)
Consider training sufficient	45 (35.7)
Consider training insufficient	81 (64.3)

While 83.3% reported being familiar with humanitarian volunteering, only 8.7% had practical experience. Approximately one quarter (26.2%) had previously experienced a disaster situation.

Overall, 61.9% of students expressed interest in working in emergency or disaster settings. However, when asked about actual availability to intervene, only 46.0% reported willingness to participate, while 31.7% were uncertain and 22.2% were unwilling.

Among those hesitant or unwilling, the main barriers were lack of personal interest (85.7%) and absence of specific training (60.7%). Conversely, adequate working conditions (26.5%) and access to specialized training (22.1%) were identified as factors that could increase willingness to engage.

Despite the relatively high interest, only 35.7% of respondents considered their undergraduate training enough to prepare them for emergency and disaster practice.

#### 3.2.3 Self-perceived competencies

##### 3.2.3.1 Interpersonal skills

Participants reported high confidence in empathy and collaboration, with 96.8 and 92.9%, respectively, rating themselves as “good” or “very good.” Resilience and adaptability were also positively evaluated. Leadership emerged as the weakest interpersonal competency, with only 57.2% reporting positive self-assessments (Table 5).

**Table 5:** Self-perceived interpersonal and technical competencies related to disaster response.

Self-perceived interpersonal competencies *n* (%)			
Competency	Poor/Very poor	Moderate	Good/Very good
Empathy	4 (3.2)	24 (19.0)	98 (77.8)
Teamwork	6 (4.8)	30 (23.8)	90 (71.4)
Resilience	14 (11.1)	42 (33.3)	70 (55.6)
Leadership	30 (23.8)	24 (19.0)	72 (57.2)

Self-perceived technical competencies *n* (%)			
Competency	Poor/Very poor	Moderate	Good/Very good
Burn management	78 (61.9)	33 (26.2)	15 (11.9)
Amputation management	74 (58.7)	38 (30.2)	14 (11.1)
Psychological first aid	69 (54.8)	36 (28.6)	21 (16.6)
Fracture immobilization	28 (22.2)	44 (34.9)	54 (42.9)
Assistive devices	34 (27.0)	41 (32.5)	51 (40.5)

##### 3.2.3.2 Technical competencies

Perceived technical competence was moderate overall, with higher confidence in areas such as fracture management, immobilization, and prescription of assistive devices. In contrast, substantial gaps were identified in domains particularly relevant to disaster contexts, including burns, amputations, and psychological first aid, where a majority of students rated themselves as poorly prepared (Table 5).

#### 3.2.4 Functional capacities in disaster response roles

Greater confidence was reported in tasks related to identifying high-risk patients (57.1% capable or very capable) and assessing environmental accessibility (48.4%). Educational interventions directed at patients and caregivers were also perceived positively (61.9% capable or very capable).

Lower levels of confidence were observed in roles requiring advanced leadership or pedagogical skills, such as training peers in specialized rehabilitation (60.3% reporting low competence), coordinating discharge and follow-up, mapping rehabilitation services, and providing psychological support and referral.

#### 3.2.5 Perception of curriculum adequacy and training needs

Most participants (64.3%) considered the curricular content insufficient to prepare them for emergency and disaster contexts. Priority areas identified for curricular reinforcement included Basic Life Support and first aid (19.0%), emergency intervention strategies (15.1%), and stress and emotional management in crisis situations (9.5%). Additional needs concerned clarification of the physiotherapist’s role in disasters, interdisciplinary collaboration, and management of acute clinical cases.

#### 3.2.6 Comparative analyses of students’ predisposition and perceived competencies by institution and academic year

Associations between students’ predisposition to work in disaster contexts and academic year were examined using chi-square tests. Comparisons were conducted within each institutional group (ESSAlcoitão vs. other institutions) between students in earlier stages of training (1st–2nd year) and those in later stages (3rd–4th year). No statistically significant differences were found in either institutional group [ESSAlcoitão: *χ*^2^(2) = 0.287, *p* = 0.866; other institutions: *χ*^2^(2) = 0.200, *p* = 0.905], suggesting that predisposition to work in disaster contexts was not associated with academic progression (Table 6).

**Table 6:** Predisposition to work in EDHA contexts by academic year (1st–2nd vs. 3rd–4th) within each institutional group.

Institution	Year of Study	No (%)	Yes (%)	Maybe (%)	Chi-square (*p*)
ESSAlcoitão	1st–2nd year *N = 47*	27.7	48.9	23.4	*χ*^2^(2) = 0.287; *p* = 0.866
	3rd–4th year *N = 46*	26.1	45.7	28.3	
Other institutions	1st–2nd year *N = 8*	12.5	37.5	50.0	*χ*^2^(2) = 0.200; *p* = 0.905
	3rd–4th year *N = 25*	8.0	44.0	48.0	

Percentages refer to the proportion of respondents within each year’s group and institution. χ^2^ = chi-square test for independence.

The questionnaire domains showed acceptable to excellent internal consistency. The results indicated acceptable reliability for the soft skills domain (*α* = 0.68) and excellent reliability for professional competencies (*α* = 0.91) and functional capacities (*α* = 0.96), supporting the aggregation of items into composite domain scores. Composite scores were calculated as the mean of the items within each domain (i.e., the sum of item scores divided by the number of items) and ranged from 1 to 5, with higher values indicating higher perceived competence.

Analysis of covariance (ANCOVA) models were conducted to examine the effect of institution (ESSAlcoitão vs. other institutions) on domain scores, while controlling for academic year. No statistically significant main effect of institution was observed for soft skills [*F*(1,123) = 1.189, *p* = 0.278, *η*^2^ = 0.010], professional competencies [*F*(1,123) = 2.718, *p* = 0.102, *η*^2^ = 0.022], or functional capacities [*F*(1,123) = 3.419, *p* = 0.067, *η*^2^ = 0.027]. Functional capacities approached statistical significance (*p* = 0.067), although the effect size remained small. Adjusted mean scores were slightly higher for students from other institutions across all domains (Table 7). Academic year showed a significant effect on professional competencies [*F*(1,123) = 63.275, *p* < 0.001, *η*^2^ = 0.340] and functional capacities [*F*(1,123) = 46.139, *p* < 0.001, *η*^2^ = 0.273], but not on soft skills [*F*(1,123) = 3.754, *p* = 0.055, *η*^2^ = 0.030], supporting its inclusion as a covariate in the models.

**Table 7:** ANCOVA results for the effect of institution (ESSAlcoitão vs. other institutions) on soft skills, professional competencies, and functional capacities, adjusted for academic year.

Dimension	ESSAlcoitão (Adjusted *M* ± SE)	Other institutions (Adjusted *M* ± SE)	*F*(1,123)	*p*	Partial *η*^2^
Soft skills	4.094 ± 0.047	4.195 ± 0.080	1.189	0.278	0.010
Professional competencies	2.356 ± 0.058	2.546 ± 0.099	2.718	0.102	0.022
Functional capacities	3.224 ± 0.086	3.539 ± 0.146	3.419	0.067	0.027

Institution (ESSAlcoitão vs. other institutions) was included as a fixed factor and academic year as a covariate in the ANCOVA models. Values represent estimated marginal means (adjusted means). *F* values correspond to the main effect of institution.

## 4 Discussion

This study contributes to the growing field of emergency, disaster, and humanitarian action by examining physiotherapy students’ predisposition, perceived preparedness, and self-assessed competencies using a validated assessment instrument. The findings offer both a methodological contribution, through the development and validation of a reliable tool, and a substantive contribution by providing empirical evidence on the current state of preparedness of future physiotherapists for disaster contexts in Portugal. The high content validity achieved through the expert consultation process, combined with positive pre-test feedback, supports the use of this questionnaire as a tool for educational evaluation, curriculum monitoring, and cross-institutional comparison. In a field where empirical measurement remains scarce, the availability of a robust instrument represents an important step toward evidence-informed educational planning. Beyond its methodological value, this study provides empirical evidence on the current state of preparedness of future physiotherapists for disaster contexts in Portugal. The findings suggest that, while students demonstrate awareness of the relevance of EDHA, important gaps remain in perceived competence, training exposure and readiness to operate in real-world disaster scenarios. These results reinforce the need for structured integration of EDHA content into undergraduate curricula and support the development of targeted educational strategies aligned with global rehabilitation and disaster response frameworks. Taken together, these findings support a clear implication: preparing physiotherapy students for emergency and disaster contexts cannot rely on motivation alone. It requires a systematic, competency-based educational strategy that integrates EDHA as a core dimension of professional identity rather than as an optional or peripheral topic. International experience suggests that such preparation is most effective when grounded in experiential learning, including simulation-based training, interprofessional education, and participation in emergency drills (32, 33). Embedding these approaches longitudinally within curricula may help transform students’ willingness to act into genuine operational readiness. Importantly, this contribution should be understood primarily within an educational and workforce pipeline perspective. The study does not aim to assess operational readiness for Emergency Medical Team (EMT) deployment, but rather to inform how undergraduate education can better prepare future physiotherapists for potential roles in emergency, disaster, and humanitarian action.

A major contribution of this research lies in the confirmation of a consistent pattern previously suggested in literature but rarely documented in physiotherapy: the coexistence of high motivation with low perceived technical preparedness. While more than one third of students reported feeling prepared to work in disaster contexts, detailed self-assessment of technical competencies revealed substantial perceived deficits, particularly in areas such as burn management, amputations, and psychological first aid, situations in which physiotherapists may contribute through rehabilitation-related interventions (e.g., functional assessment, positioning, early mobilization, prevention of complications, and support to recovery), rather than to the performance of acute medical or surgical procedures (34).

This apparent contradiction reflects a dissociation between global perceived readiness and domain-specific competence, a phenomenon increasingly described as a *readiness–competence gap* in disaster preparedness research (14, 17, 18). Rather than indicating overconfidence, this pattern suggests that students can critically distinguish between their willingness to engage and their actual operational capacity. This distinction further reinforces that the present findings reflect an educational stage of professional development, rather than criteria for clinical or operational deployment in emergency settings. In addition to its descriptive value, the assessment tool developed in this study may serve as a practical instrument to support curriculum development in physiotherapy education. By identifying specific domains in which students report lower perceived competences such as psychological first aid, management of complex trauma, and leadership in disaster context the questionnaire can help educators detect gaps in current training and prioritize targeted educational interventions. For example, results may inform the integration of simulation-based learning, interdisciplinary training, disaster preparedness modules, and structured field exercises aimed at strengthening both technical and non-technical competencies relevant to emergency, disaster, and humanitarian action contexts. In this way, the instrument may contribute not only to monitoring students’ preparedness but also to guiding educational strategies that progressively build competencies in disaster rehabilitation (32, 35).

Although this study was not originally designed within a specific theoretical framework, the observed dissociation between willingness and perceived competence may be interpreted through the lens of self-efficacy theory (36). Students may maintain strong motivational beliefs and professional commitment while simultaneously recognizing limited efficacy in specific tasks when experiential learning opportunities are scarce. This interpretation aligns with the results of the present study, in which students demonstrated high levels of altruism, empathy, and collaboration, yet expressed low confidence in technical and leadership-related domains. Such a profile highlights that disaster readiness is not a unidimensional construct, but rather the product of interacting motivational, emotional, and technical components.

The strong interest in EDHA contexts observed among participants echoes findings from international studies involving health professions students, which consistently report high levels of social responsibility and willingness to contribute to crises (13, 16). However, as in other educational contexts, this motivation is not supported by a corresponding perception of adequate preparation. Only a minority of respondents considered their undergraduate training enough for disaster practice, reinforcing concerns that EDHA remains marginal in most physiotherapy curricula. Similar findings have been reported in other countries. For example, a national survey of physiotherapy training institutions in Japan found that only a limited proportion of programs offer formal teaching on disaster rehabilitation, with curriculum overload and lack of qualified instructors identified as major barriers to implementation (15). This is particularly concerning given the growing recognition of rehabilitation as an essential pillar of emergency response systems, with physiotherapists now formally integrated into international Emergency Medical Teams (3, 5, 34). While this institutional recognition underscores the relevance of disaster preparedness in physiotherapy, it should be noted that EMT deployment standards apply to licensed and experienced professionals. In contrast, the present study focuses on students and therefore contributes primarily to understanding how educational systems can build the future rehabilitation workforce pipeline for such roles.

A distinctive contribution of this study is the detailed mapping of which competencies students perceive as fragile. Beyond identifying general unpreparedness, the findings specify concrete gaps in psychological first aid, management of complex trauma, and leadership. These results extend previous work by moving beyond generic calls for “more disaster education” and providing actionable directions for curriculum development. They suggest that strengthening disaster preparedness in physiotherapy requires not only technical upskilling, but also intentional development of non-technical skills, particularly leadership, decision-making under pressure, and interprofessional coordination, competencies consistently highlighted in international EDHA guidelines (22, 23).

The analysis of functional roles further reinforces this point. Students expressed greater confidence in tasks that overlap with conventional clinical practice, such as patient education, environmental assessment, and risk identification. In contrast, system-level functions – training peers, coordinating care pathways, mapping rehabilitation services, and managing follow-up – were associated with markedly lower self-efficacy. This pattern indicates that current training may be effective in fostering individual clinical competence, but insufficient in preparing students for the organizational and leadership demands of disaster response. Such imbalance is problematic, as EDHA practice requires professionals who can operate not only at the bedside but also at the level of service organization and community coordination.

Another relevant finding concerns the absence of significant differences in predisposition between students in earlier and later academic years. This suggests that willingness to engage in disaster contexts may be shaped more by personal values and attitudes than by formal progression through the academic curriculum. This interpretation becomes particularly meaningful when considering the curricular context of the participating institutions. Notably, none of the six programs included in this sample offer a dedicated, mandatory curricular unit focused on emergency, disaster, and humanitarian action preparedness. When these topics are addressed, they are typically covered in a sporadic and fragmented manner across different courses, without a formal, longitudinal, or competency-assessed structure. Consequently, the lack of a clear developmental trend in predisposition across academic years can likely be attributed to this absence of systematic and progressive educational exposure. This stands in clear contrast to evidence showing that perceived competence in disaster response increases following structured and targeted training (32, 35). The lack of a clear developmental trend in the present study may therefore reflect the limited and unsystematic integration of EDHA content in undergraduate physiotherapy programs, resulting in minimal cumulative impact across academic years.

This study has limitations that should be acknowledged. The reliance on self-reported data may introduce social desirability bias, particularly in the assessment of interpersonal skills. Cross-sectional design prevents analysis of developmental trajectories or causal relationships between training exposure and preparedness. Moreover, because the sample consisted exclusively of pre-licensure students, the findings cannot be extrapolated to EMT deployability or compliance with international deployment standards for rehabilitation professionals. Instead, they should be interpreted as informing educational preparedness and long-term capacity building in emergency, disaster, and humanitarian action. Additionally, the uneven distribution of participants across institutions limits the generalizability of institutional comparisons. Future research should adopt longitudinal designs to track changes in preparedness across the training continuum and incorporate qualitative approaches to explore students’ motivations, fears, and perceived barriers in greater depth.

Despite these limitations, this study adds meaningful evidence to an underexplored area of physiotherapy education. By documenting the coexistence of strong motivation with perceived technical insufficiency, it highlights both a challenge and an opportunity: a generation of future physiotherapists eager to contribute in crises, whose potential can be realized through intentional educational investment and progressive workforce development. Addressing this gap through curriculum reform and targeted capacity-building may not only strengthen professional development but also enhance the resilience of health systems in the face of increasingly frequent humanitarian and disaster scenarios.

## 5 Conclusion

This study contributes to emergency, disaster, and humanitarian action (EDHA) education in physiotherapy by developing and validating an instrument that assesses students’ predisposition, perceived competence, and readiness to assume functional roles in disaster contexts. The tool provides a structured way to characterize professional preparedness in an area where empirical assessment remains limited. The findings reveal a consistent readiness–competence gap: although students demonstrate strong motivation to engage in EDHA, they report limited confidence in several technical and organizational competencies required for practice, suggesting that current undergraduate training provides insufficient exposure to disaster-related competencies.

These results highlight the need for the systematic integration of EDHA within physiotherapy education, ensuring that students’ willingness to contribute in crisis settings is supported by appropriate competencies. The instrument presented in this study may serve as a strategic resource for guiding and evaluating curriculum development, supporting the preparation of a more competent rehabilitation workforce and strengthening health system preparedness for future emergencies and disasters. Additionally, the questionnaire may serve as a monitoring tool for evaluating the impact of future educational interventions and curriculum changes aimed at strengthening EDHA preparedness among physiotherapy students.

## Data Availability

The original contributions presented in the study are included in the article/Supplementary material, further inquiries can be directed to the corresponding author.
