# Supplementary material for: Measuring readiness for disaster response in physiotherapy education: development and validation predisposition assessment tool
Source: Front Public Health. 2026 Jun 1;14:1814454. doi: 10.3389/fpubh.2026.1814454 (PMC13265569; doi:10.3389/fpubh.2026.1814454)
Supplement: Supplementary file 1 [file Data_Sheet_1.pdf]

Supplementary File 1
Preliminary Version of the Questionnaire

This preliminary Portuguese version corresponds to the initial item pool developed during the instrument construction phase. It was used exclusively for content validation through expert panel consultation. This version was not used for data collection. The final validated version of the questionnaire is presented in **Supplementary File 2**.

Predisposição dos estudantes de fisioterapia para trabalhar em contexto de catástrofe

Contexto de Catástrofe

1. Tem conhecimento da probabilidade de catástrofe na sua área residencial?

   ☐ Sim ☐ Não

2. Tem conhecimento do plano de catástrofe da sua área de residência/município?

   ☐ Sim ☐ Não

3. Tem interesse em trabalhar enquanto fisioterapeuta em contextos de emergência, catástrofe ou ação humanitária?

   ☐ Sim ☐ Não

4. Porquê?

   ☐ Considero não ter competências técnicas (hard skills) e/ou interpessoais (soft skills) adequadas ao contexto

   ☐ Outros

5. Se respondeu outro, qual o motivo?

   ____

Competências interpessoais e técnicas em contexto de catástrofe

Sendo o contexto de catástrofe um contexto muito específico, é necessário a aquisição de algumas competências interpessoais (soft skills) e competências técnicas e profissionais (hard skills) para atuar no mesmo.

6. Como se autoavalia nas seguintes soft skills?

	Muito pobre	***Pobre***	***Moderado***	***Bom***	***Muito bom***
Altruísmo	☐	☐	☐	☐	☐
Adaptação	☐	☐	☐	☐	☐
Liderança	☐	☐	☐	☐	☐
Colaboração	☐	☐	☐	☐	☐
Resiliência	☐	☐	☐	☐	☐
Empatia	☐	☐	☐	☐	☐

7. Como autoavalia as suas competências técnicas para vir a desempenhar funções de fisioterapeuta nas seguintes áreas de intervenção?

	Nada competente	***Pouco competente***	***Moderadamente competente***	***Competente***	***Muito competente***
Lesões vértebro-medulares	☐	☐	☐	☐	☐
Lesões neurais	☐	☐	☐	☐	☐
Fraturas	☐	☐	☐	☐	☐
Lesões neuromusculares	☐	☐	☐	☐	☐
Queimaduras, enxertos e retalhos	☐	☐	☐	☐	☐
Amputações	☐	☐	☐	☐	☐
Fisioterapia cardiorrespiratória	☐	☐	☐	☐	☐
Imobilização com talas	☐	☐	☐	☐	☐
Prescrição de cadeira de rodas	☐	☐	☐	☐	☐
Primeiros socorros psicológicos	☐	☐	☐	☐	☐

Inclusão da deficiência	☐	☐	☐	☐	☐

8. Segundo a World Physio o fisioterapeuta apresenta diferentes papeis em contexto de catástrofe sendo alguns para além do corpo de saberes do fisioterapeuta. Como autoavalia as suas capacidades nos seguintes papeis?

	Nada capaz	***Pouco capaz***	***Razoável***	***Capaz***	***Muito capaz***
Avaliar a necessidade dereabilitação numa situação dedesastre	☐	☐	☐	☐	☐
Mapear os serviços dereabilitação disponíveis	☐	☐	☐	☐	☐
Providenciar reabilitação emsituações agudas em hospitaislocais e na comunidade	☐	☐	☐	☐	☐
Providenciar educação aos utentes, cuidadores e outrosprofissionais de saúde	☐	☐	☐	☐	☐
Realizar triagem e referenciaras vítimas	☐	☐	☐	☐	☐
Coordenar as altas, referenciação e follow-up	☐	☐	☐	☐	☐
Providenciar apoio psicológico e referenciar para serviços indicados	☐	☐	☐	☐	☐
Avaliar, prescrever, ajustar e providenciar produtos de apoio e respetivo treino e manutenção	☐	☐	☐	☐	☐
Avaliar as condições ambientais e a necessidade de adaptação para tornar as áreas acessíveis	☐	☐	☐	☐	☐
Identificar utentes de maior risco	☐	☐	☐	☐	☐
Providenciar cuidados de prevenção e reabilitação para idosos e utentes com condições crónicas afetados pelo desastre	☐	☐	☐	☐	☐
Treinar colegas em reabilitação em contextos mais específicos como amputações ou lesões vertebro-medulares	☐	☐	☐	☐	☐
Treinar colegas para identificar e reencaminhar pessoas com necessidade de reabilitação	☐	☐	☐	☐	☐

Predisposição

9. Estaria predisposto a trabalhar em contexto de catástrofe?
   ☐ Sim ☐ Não ☐ Talvez

10. Se não, qual o principal motivo?

    ☐ Remuneração

    ☐ Demasiado tempo longe de casa

    ☐ Familiares dependentes

    ☐ Não tenho interesse na área

    ☐ Outro; Qual? ____

11. Identifique a(s) condicionante(s) que faria com que estivesse disponível para trabalhar neste contexto.

    ____

12. Considera que os conteúdos programáticos abordados ao longo da licenciatura são os necessários para capacitar o fisioterapeuta para trabalhar neste contexto?
    ☐ Sim ☐ Não

13. Que conteúdos adicionais considera que seria necessário abordar?

    ____

Terminou o questionário

Muito obrigada pela sua colaboração!

Disclaimer regarding the English translation

This English translation is provided for informational purposes only to support readers' comprehension of the original Portuguese instrument. It does not constitute a validated version. Any use of this questionnaire in other linguistic or cultural contexts requires prior permission from the authors and must follow established guidelines for translation, cross-cultural adaptation, and validation.

Predisposition of physiotherapy students to work in the context of a disaster

Disaster Context Awareness

1. Do you have knowledge about the probability of disaster occurrence in your residential area?

   ☐ Yes ☐ No

2. Are you aware of the disaster response plan of your municipality or residential area?

   ☐ Yes ☐ No

3. Are you interested in working as a physiotherapist in emergency, disaster, or humanitarian action contexts?

   ☐ Yes ☐ No

4. Why?

   ☐ Lack of technical competencies (hard skills) and/or interpersonal (soft skills) appropriate to the context

   ☐ Other

5. If you answered "Other", what is the reason: ______________________________

Interpersonal and technical skills in the context of a disaster

As the context of a disaster is a very specific context, it is necessary to acquire some interpersonal skills (soft skills) and technical and professional skills (hard skills) to act in it.

6. How do you rate your level in the following interpersonal competencies (soft skills)?

	Very poor	***Poor***	***Moderate***	***Good***	***Very good***
Altruism	☐	☐	☐	☐	☐
Adaptability	☐	☐	☐	☐	☐
Leadership	☐	☐	☐	☐	☐
Collaboration	☐	☐	☐	☐	☐
Resilience	☐	☐	☐	☐	☐
Empathy	☐	☐	☐	☐	☐

7. How do you self-assess your technical skills to perform physiotherapist functions in the following areas of intervention?

	Not competent	***Slightly competent***	***Moderately competent***	***Competent***	***Highly competent***
Spinal cord injuries	☐	☐	☐	☐	☐
Neurological injuries	☐	☐	☐	☐	☐
Fractures	☐	☐	☐	☐	☐
Neuromuscular conditions	☐	☐	☐	☐	☐
Burns and graft management	☐	☐	☐	☐	☐
Amputations	☐	☐	☐	☐	☐
Cardiorespiratory physiotherapy	☐	☐	☐	☐	☐
Immobilisation techniques	☐	☐	☐	☐	☐
Wheelchair prescription	☐	☐	☐	☐	☐
Psychological first aid	☐	☐	☐	☐	☐
Disability inclusion	☐	☐	☐	☐	☐

According to World Physio, the physiotherapist has different roles in the context of catastrophe, some of which are beyond the body of knowledge of the physiotherapist.

8. How do you self-assess your abilities in the following roles**?**

	Not capable	***Slightly capable***	***Reasonably capable***	***Capable***	***Highly capable***
Assess rehabilitation needs in disaster situations	☐	☐	☐	☐	☐
Map available rehabilitation services	☐	☐	☐	☐	☐
Provide rehabilitation in acute contexts	☐	☐	☐	☐	☐
Educate patients, caregivers, and professionals	☐	☐	☐	☐	☐
Perform triage and refer victims	☐	☐	☐	☐	☐
Coordinate discharge and follow-up	☐	☐	☐	☐	☐
Provide psychological support and referral	☐	☐	☐	☐	☐
Prescribe and train use of assistive products	☐	☐	☐	☐	☐
Assess environmental accessibility needs	☐	☐	☐	☐	☐
Identify individuals at higher functional risk	☐	☐	☐	☐	☐
Provide care to older adults and chronic patients affected by disasters	☐	☐	☐	☐	☐
Train peers in specialised rehabilitation (e.g., amputations, spinal injuries)	☐	☐	☐	☐	☐
Train peers to identify rehabilitation needs	☐	☐	☐	☐	☐

Predisposition

9. Would you be willing to work in disaster contexts?

   ☐ Yes ☐ No ☐ Maybe

10. If not, what is the main reason?

    ☐ Financial conditions ☐ Time away from home ☐ Family responsibilities

    ☐ Lack of interest ☐ Other; What? ______________________________________

11. Identify the condition(s) that would make you available to work in this context.

    ____

12. Do you consider that the syllabus covered throughout the degree is necessary to enable the physiotherapist to work in this context?

    ☐ Yes ☐ No

13. What additional contents should be included in the curriculum?

    ____

You finished the questionnaire.

Thank you very much for your cooperation!
