# Supplementary material for: Measuring readiness for disaster response in physiotherapy education: development and validation predisposition assessment tool
Source: Front Public Health. 2026 Jun 1;14:1814454. doi: 10.3389/fpubh.2026.1814454 (PMC13265569; doi:10.3389/fpubh.2026.1814454)
Supplement: Supplementary file 2 [file Data_Sheet_2.pdf]

Predisposição dos Estudantes de Fisioterapia para Trabalhar em Contexto de Catástrofe, Emergência e Ação Humanitária

Instruções: As questões que se seguem estão divididas em três secções em que lhe pedem a opinião sobre a sua predisposição para trabalhar como fisioterapeuta em contexto de emergência, catástrofe, e ação humanitária, a forma como se sente e sobre a sua capacidade de desempenhar as atividades habituais. Pedimos que leia com atenção cada pergunta e que responda o mais honestamente possível. Se não tiver a certeza sobre a resposta a dar, dê-nos a que considerar mais apropriada.

Secção 1: Contexto de catástrofe – Por favor, assinale a resposta mais adequada.

1.Tem conhecimento se a sua área residencial é uma zona de risco?

□ Sim

□ Não

2. Conhece o plano de catástrofe da sua área de residência/município?

□ Sim

□ Não

3. Sabe como ter acesso ao plano de catástrofe?

□ Sim

□ Não

4. Está em desenvolvimento em Portugal uma nova área de abordagem do fisioterapeuta em contexto de emergência, catástrofe e ação humanitária. Tem interesse em trabalhar em algum destes contextos?

□ Sim

□ Não

a. Se escolheu que não, qual/quais o(s) motivo(s)? (Selecione todas as opções aplicáveis)

i. Considero não ter competências técnicas (*hard skills*) adequadas ao contexto

ii. Considero não ter competências interpessoais (*soft skills*) adequadas ao contexto

iii. Não tenho interesse nesta área

iv. Não vejo a importância do fisioterapeuta nesta área

v. Outro

b. Se respondeu outro, qual o motivo?

____

Copyright@2025. Todos os direitos reservados. Permissão necessária para reprodução ou uso.

Secção 2: Competências interpessoais e técnicas em contexto de catástrofe – Por favor, assinale a resposta mais adequada.

Sendo o contexto de catástrofe um contexto muito específico, é necessário a aquisição de algumas competências interpessoais (*soft skills*) e competências técnicas e profissionais (*hard skills*) para atuar no mesmo.

5. Como se autoavalia nas seguintes *soft skills*?

	muito pobre	pobre	moderado	bom	muito bom
Altruísmo					
Adaptação					
Liderança					
Colaboração					
Resiliência					
Empatia					

6. Como autoavalia as suas competências profissionais para vir a desempenhar funções de fisioterapeuta nas seguintes áreas de intervenção?

	nada competente	pouco competente	moderadamente competente	competente	muito competente
Lesões medulares					
Lesões cerebrais					
Fraturas					
Lesões do sistema nervoso periférico					
Queimaduras, enxertos e retalhos					
Amputações					
Condições Cardiorrespiratória					
Imobilização					
Prescrição de produtos de apoio					
Primeiros socorros psicológicos					
Inclusão da deficiência					

Copyright@2025. Todos os direitos reservados. Permissão necessária para reprodução ou uso.

7. Segundo a *World Physiotherapy* o fisioterapeuta apresenta diferentes papeis em contexto de catástrofe sendo alguns para além do corpo de saberes do fisioterapeuta. Como autoavalia as suas capacidades nos seguintes papeis?

	nada capaz	pouco capaz	moderadamente capaz	capaz	muito capaz
Avaliar a necessidade de reabilitação numa situação de desastre					
Mapear os serviços de reabilitação disponíveis					
Providenciar reabilitação em situações agudas em hospitais locais e na comunidade					
Providenciar educação aos utentes, cuidadores e outros profissionais de saúde					
Realizar triagem e referenciaras vítimas					
Coordenar as altas, referenciação e follow-up					
Providenciar apoio psicológico e referenciar para serviços indicados					
Avaliar, prescrever, ajustar e providenciar produtos de apoio e respetivo treino e manutenção					
Avaliar as condições ambientais e a necessidade de adaptação para tornar as áreas acessíveis.					
Identificar utentes de maior risco					
Providenciar cuidados de prevenção e reabilitação para idosos e utentes com condições crónicas afetados pelo desastre					
Treinar colegas em reabilitação em contextos mais específicos como amputações ou lesões vertebro-medulares					
Treinar colegas para identificar e reencaminhar pessoas com necessidade de reabilitação					

Copyright@2025. Todos os direitos reservados. Permissão necessária para reprodução ou uso.

Secção 3: Predisposição – Por favor, assinale a resposta mais adequada.

8. Estaria disposto a trabalhar em contexto de catástrofe?

□ Sim

□ Não

□ Talvez

9. Se escolheu não ou talvez, quais os principais motivos? (Selecione todas as opções aplicáveis)

□ Condições de remuneração

□ Longos períodos deslocado

□ Familiares dependentes

□ Condições de segurança

□ Falta de formação

□ Falta de informação relativa ao papel do fisioterapeuta

□ Falta de interesse na área

□ Outro

a. Se respondeu "outro", qual/quais o(s) motivo(s)?

____

10. Identifique a(s) condição(ões) que faria(m) com que estivesse disponível para trabalhar neste contexto.

____

____

____

11. Considera que os conteúdos programáticos abordados ao longo da licenciatura são os necessários para habilitar o fisioterapeuta para trabalhar neste contexto?

□ Sim

□ Não

12. Quais são os conteúdos adicionais que considera importante serem abordados?

____

____

Muito obrigado(a) pela sua colaboração!

Copyright@2025. Todos os direitos reservados. Permissão necessária para reprodução ou uso.

Note regarding the English translation of the questionnaire

The questionnaire was originally developed in Portuguese. The English translation presented below is provided solely to facilitate international readers' understanding of the instrument content.
This translation has not undergone a formal process of forward–backward translation, cross-cultural adaptation, or psychometric validation. Therefore, it should not be used for research, educational, or clinical purposes without prior authorization from the authors and without following established methodological procedures for translation and validation of measurement instruments.
Unauthorized reproduction, adaptation, or use of this instrument without the authors' consent is not permitted.

Physiotherapy Students' Predisposition to Work in Disaster, Emergency and Humanitarian Action Contexts

Instructions: *The following questions are divided into three sections where you are asked for your opinion on the their predisposition to work as a physiotherapist in the context of emergency, catastrophe, and humanitarian action, the way they feel and about their ability to perform usual activities. We ask that you read each question carefully and answer as honestly as possible. If you are unsure about the answer to give, please give us the one you consider most appropriate.*

Section 1: Disaster context – Please tick the most appropriate response.

1. *Do you know if your residential area is a risk zone?*

   □ *Yes*

   □ *No*

2. *Do you know the disaster plan for your area of residence/municipality?*

   □ *Yes*

   □ *No*

3. *Do you know how to access the disaster plan?*

   □ *Yes*

   □ *No*

4. *A new area of approach for physiotherapists in the context of emergency, catastrophe and humanitarian action is being developed in Portugal. Are you interested in working in any of these contexts?*

   □ *Yes*

   □ *No*

   a. *If you chose no, what is the reason(s)? (Select all that apply)*

      i. *I consider that I do not have technical skills (hard skills) appropriate to the context*

      ii. *I consider that I do not have interpersonal skills (soft skills) appropriate to the context*

      iii. *I have no interest in this area*

      iv. *I don't see the importance of the physiotherapist in this area*

      v. *Other*

   b. *If you answered Other, what is the reason?*

   ____

Copyright@2025. All rights reserved. Permission required for reproduction or use.

Section 2: Interpersonal and technical skills in the context of a disaster – Please tick the most appropriate answer.

As the context of a disaster is a very specific context, it is necessary to acquire some interpersonal skills (soft skills) and technical and professional skills (hard skills) to act in it.

5. *How do you self-assess in the following soft skills?*

	Very poor	Poor	Moderate	Good	Very good
Altruism					
Adaptation					
Leadership					
Collaboration					
Resilience					
Empathy					

6. *How do you self-assess your professional skills to perform physiotherapist functions in the following areas of intervention?*

	Not at all competent	Slightly competent	Moderately competent	Competent	Very competent
Spinal cord injuries					
Brain injuries					
Fractures					
Peripheral nervous system injuries					
Burns, grafts and flaps					
Amputations					
Cardiorespiratory Conditions					
Immobilization					
Prescription of support products					
Psychological first aid					
Disability inclusion					

Copyright@2025. All rights reserved. Permission required for reproduction or use.

7. *According to World Physiotherapy, the physiotherapist has different roles in the context of catastrophe, some of which are beyond the body of knowledge of the physiotherapist. How do you self-assess your skills in the following roles?*

	Not capable at all	*Slightly capable*	*Moderately capable*	*Capable*	*Very capable*
Assessing the need for rehabilitation in a disaster situation					
Map available rehabilitation services					
Provide acute rehabilitation in local hospitals and in the community					
Provide education to patients, caregivers and other health professionals					
Perform triage and refer victims					
Coordinate discharges, referral and follow-up					
Provide psychological support and referral to indicated services					
Assess, prescribe, adjust, and provide support products and their training and maintenance					
Assess environmental conditions and the need for adaptation to make areas accessible.					
Identify patients at higher risk					
Provide preventive and rehabilitative care for the elderly and patients with chronic conditions affected by the disaster					
Train colleagues in rehabilitation in more specific contexts such as amputations or spinal cord injuries					
Train colleagues to identify and refer people in need of rehabilitation					

Copyright@2025. All rights reserved. Permission required for reproduction or use.

Section 3: Predisposition – Please tick the most appropriate answer.

8. *Would you be willing to work in the context of a catastrophe?*

   - ☐ *Yes*
   - ☐ *No*
   - ☐ *Maybe*

9. *If you chose No or Maybe, what are the main reasons? (Select all that apply)*

   - ☐ *Conditions of remuneration*
   - ☐ *Long periods displaced*
   - ☐ *Dependent family members*
   - ☐ *Safety conditions*
   - ☐ *Lack of training*
   - ☐ *Lack of information regarding the role of the physiotherapist*
   - ☐ *Lack of interest in the area*
   - ☐ *Other*

a. *If you answered "Other", what is the reason(s)?*

   ____

10. *Identify the condition(s) that would make you available to work in this context.*

   ____

   ____

   ____

11. *Do you consider that the syllabus covered throughout the degree is necessary to enable the physiotherapist to work in this context?*

   - ☐ *Yes*
   - ☐ *No*

12. *What additional content you consider important to cover?*

   ____

   ____

Thank you very much for your cooperation!

Copyright@2025. All rights reserved. Permission required for reproduction or use.
